# Functional Enrichment and Analysis of Antigen-Specific Memory B Cell Antibody Repertoires in PBMCs

**DOI:** 10.3389/fimmu.2019.01452

**Published:** 2019-06-25

**Authors:** Eric Waltari, Aaron McGeever, Natalia Friedland, Peter S. Kim, Krista M. McCutcheon

**Affiliations:** ^1^Chan Zuckerberg Biohub, San Francisco, CA, United States; ^2^Stanford ChEM-H and Department of Biochemistry, Stanford University School of Medicine, Stanford, CA, United States

**Keywords:** antibody, repertoire, sequencing, memory B cell, PBMC, clonal families

## Abstract

Phenotypic screening of antigen-specific antibodies in human blood is a common diagnostic test for infectious agents and a correlate of protection after vaccination. In addition to long-lived antibody secreting plasma cells residing in the bone marrow, memory B cells are a latent source of antigen-experienced, long-term immunity that can be found at low frequencies in circulating peripheral blood mononuclear cells (PBMCs). Assessing the genotype, clonal frequency, quality, and function of antibodies resulting from an individual's persistent memory B cell repertoire can help inform the success or failure of immune protection. Using *in vitro* polyclonal stimulation, we functionally expand the memory repertoire from PBMCs and clonally map monoclonal antibodies from this population. We show that combining deep sequencing of stimulated memory B cell repertoires with retrieving single antigen-specific cells is a promising approach in evaluating the latent, functional B cell memory in PBMCs.

## Introduction

One of public health's most cost-effective interventions to prevent and reduce the disease burden of infectious disease is vaccination. It is well-established that immunological memory stored in antigen-specific B cell repertoires frequently forms the foundation of successful natural or vaccine-induced immune protection (1). During the acute phase of pathogen exposure, naïve B cells enter germinal centers and undergo immunoglobulin (Ig) receptor somatic hypermutation (SHM) to increase antigen binding, isotype class switching to IgG or IgA for effector activities, and differentiation to short-lived antibody secreting plasmablasts or to long-lived plasma and memory B cells (2). Successful diversification of B cell clones and their corresponding Ig receptors creates clonal families, each a cluster or lineage of related antibodies all descended from the naïve B cell ancestor (3), and possessing variations in antigen binding and functional activities. Antibodies secreted from plasma cells, which mainly reside in the bone marrow, provide steady state protection against repeat infections. In contrast, memory B cells survive in a functionally quiescent state in tissues and can be found at low frequency in PBMCs after a pathogen is eliminated, re-activating and differentiating into antibody secreting plasmablasts within a week of a secondary infection (4–6). Evaluating both types of adaptive B-cell memory is challenged by the anatomical inaccessibility and rarity of these cells. A variety of serological assays for polyclonal antibody measurements have been developed (7) and, more recently, the applications of next generation sequencing (NGS) and single cell sequencing technologies have offered high resolution data on B cell antibody responses in peripheral blood samples (8). Computational algorithms to interpret genetic relationships between the millions of NGS B cell receptor (BCR) sequences have been developed to assign clonal families and help reconstruct *in vivo* B cell clonal lineages (9). However, outside of the acute phase of exposure, the depth of antigen-experienced B cell repertoires is still limited by the low frequency and relative transcriptional silence of memory B cells circulating in human blood.

The maintenance of memory B cells *in vivo* in the absence of persisting antigen has been proposed to occur through polyclonal stimulation mechanisms including: (i) Toll-like receptor (TLR) microbial agonists such as lipopolysaccharide (TLR4 agonist) or unmethylated single-stranded DNA motifs (e.g., CpG-B, a TLR9 agonist); and (ii) T cell bystander activation via CD40 ligation and cytokine production (10). When memory B cells are polyclonally activated *in vitro* independent of BCR signals, they proliferate and differentiate to plasma cells while maintaining specific antigen binding (10). There is no direct evidence excluding SHM in the BCRs using this technique, and the optimal combination of microbial products, cytokines, and feeder cells determined varies between labs (10–12). However, polyclonal stimulation of PBMCs is frequently applied with ELISPOT techniques to quantitate the total number of cells secreting IgG and report percentages of antigen-specific cells in response to vaccination. The specificity of the *in vitro* response for memory B cells has been supported by (i) the retention of signal to the relevant antigen and lack of signal to an irrelevant antigen; (ii) the absence of antigen-specific cell spots in unvaccinated, naïve blood donor PBMCs; and, (iii) positive ELISPOTs from cultures where CD19^+^CD20^+^ B cells were first depleted from PBMC and reconstituted with CD27^+^ (memory), but not CD27^−^ (naïve), B cells. Furthermore, broadly cross-reactive binding and neutralizing recombinant antibodies directed to Influenza A and HCMV from polyclonally stimulated PBMCs have been cloned from single B cells using this method (12).

Herein, we show the benefit of combining NGS with *in vitro* cell culture methods that functionally and selectively expand memory B cells within PBMC samples using CpG-B stimulation and cytokines. Using three healthy donors of different genetic backgrounds we compared Ig NGS data of PBMC repertoires with stimulated PBMC repertoires. In stimulated PBMCs we observed an enrichment of sequences of IgG subtypes and higher rates of SHM with no resulting bias in the representation of heavy or light chain variable domain families. We identified common V-D-J BCR sequences within one donor that persisted over three blood draws spanning a 9-month period, in PBMC and in stimulated PBMC Ig repertoires. The majority of the persistent BCR sequences identified in the stimulated repertoire, while fewer in number, showed >1% SHM and isotype switching to IgG, consistent with being part of an antigen-experienced memory population. From the same donor, we cloned antigen-specific monoclonal antibodies (mAbs) from single memory B cells isolated by FACS with the hemagglutinin trimer of influenza A H1N1. Members of these mAbs clonal lineages were identified in PBMCs vs. stimulated PBMCs repertoires, quantified, and tracked over the three blood draws. An increased sensitivity for detection of individual mAbs along with larger, branching clonal families were obtained using data from stimulated PBMCs. Overall, our data support the application of stimulating PBMC samples to gain a deeper understanding of antigen-specific circulating memory B cell repertoires.

## Materials and Methods

### Isolation of PBMCs

Leukapheresis was performed on three normal donors using Institutional Review Board (IRB)-approved consent forms and protocols by StemCell Technologies (Vancouver, BC). Approximately two to three blood volumes were processed using the Spectra Optia® Apheresis System to produce a full-sized Leuko Pak. Acid citrate dextrose, solution A (ACDA) was the anticoagulant. Donors included: 536, African American age 33, male, 82 kg, 180 cm, smoker, collected 11/15/2017, 5/24/2018, and 08/27/2018; 147, Caucasian female age 21, 56 kg, 153 cm, non-smoker, collected 08/21/2018; and 682, Hispanic male age 30, 74 kg, 181 cm, smoker, collected 08/16/2018. PBMCs were further enriched from the buffy coats of 50 ml tubes with 25 ml sterile Histopaque 1.077 (MilliporeSigma, Burlington, MA) layered with 25 ml leukocytes diluted 1:1 in PBS (no Ca^++^/Mg^++^), after centrifuging at 400 × g, 20°C, for 50 min (Eppendorf Centrifuge 5810, with swinging bucket rotor S-4-104, brake off). PBMCs from each 50 ml tube were washed by diluting three times with 50 ml ambient temperature RPMI 1640 and centrifuged 200 × g, 20°C, 10 min. PBMCs were cryopreserved in 90% heat-inactivated FBS, 10% DMSO at 1 × 10^7^ cells per vial.

### Preparation of Growth-Arrested Feeder Cells

Human fibroblast cell line MRC-5 (CCL-171) was obtained from ATCC® (Manassas, VA) and grown in B cell growth media containing Corning® DMEM [+] 4.5 g/L glucose, sodium pyruvate [–] L-glutamine (VWR International, Radnor, PA), 1xPen/Strep/Glu and 10% ultralow IgG HI-FBS (Thermo Fisher Scientific, Waltham, MA), to 80% confluence before being treated for 4 h with 5 μg/ml mitomycin C (Tocris, R&D Systems). Monolayers of growth arrested cells were washed 3 times with PBS, harvested with trypsin, neutralized with growth media, washed 1x in growth media and finally cryopreserved using 10% DMSO, 30% HI-FBS in growth media.

### Preparation of PBMCs for BCR Repertoire Analysis

PBMC vials were thawed rapidly in a 37°C water bath, immediately diluted into 10 ml of B cell growth media, and pelleted at 350 × g for 5 min. The sample was resuspended in growth media and B cells were negatively selected using the EasySep Human B cell Isolation Kit according to the manufacturer's protocol (STEMCELL Technologies, Vancouver, BC). Because our samples contained greater than one million PBMCs, B cell enrichment was used to provide a corresponding enrichment of B cell RNA in the total RNA added to the reverse transcription reaction. The final cells from negative enrichment were pelleted at 350 × g for 5 min, resuspended and lysed in Qiagen RLT buffer with beta-mercaptoethanol for 10 min, frozen on dry ice, and transferred to −80°C storage until RNA purification with the Qiagen AllPrep RNA/DNA kit (Qiagen, Hilden, Germany).

For generating stimulated PBMC samples, we applied methods previously published by McCutcheon et al. (12) to clone broadly neutralizing single B cells. The day before PBMCs were thawed, 1.2 × 10^6^ feeder cells were seeded in a T25 flask (VWR) in 4 ml of B cell growth media and cultured overnight in a humidified 37°C, 5% CO_2_ incubator. PBMCs were quickly thawed at 37°C, washed 1x in 10 ml of growth media, and resuspended in 4 ml of 2x B cell growth media containing 2 × ITS from 100x Insulin, Transferrin, Selenium (Thermo Fisher Scientific), 20 ng/ml IL-10, 2 ng/ml IL-2, 10 ng/ml IL-15, 10 ng/ml IL-6 (R&D Systems, Minneapolis, MN) and 4 μg/ml CpG (ODN 2006-G5, InvivoGen, San Diego, CA). The 4 ml of PBMCs were then added to the T25 flask with 4 ml of conditioned feeder cell media. The final 8 ml cell culture was allowed to grow for 5 days at 37°C, 5% CO_2_ in a humidified incubator. At day 5 the cells were pelleted at 350 × g for 5 min, resuspended and lysed in Qiagen RLT buffer with beta-mercaptoethanol for 10 min, frozen on dry ice, and transferred to −80°C storage until RNA purification. Since the culture conditions specifically expand memory B cells at the expense of viability of other cell types, no B cell enrichment was utilized at this step. Supernatants were also harvested to assay total IgG, IgA, and IgM secretion by indirect ELISA, as described below.

### Indirect ELISA Measurement of Total IgG, IgA, and IgM

96-well, Nunc Maxisorp™ plates (VWR, Radnor, PA) were coated overnight with anti-human IgG Fc (Jackson ImmunoResearch, West Grove, PA), anti-human IgA Fc or anti-human IgM Fc (Bethyl Labs, Montgomery, TX) at 2 μg/ml in PBS, pH7.2. The next day the plate was washed 3x 300 μl PBST and blocked for 1 h in 1% BSA/PBS. A standard curve was prepared using human reference serum (Bethyl Labs) in 1/3 dilutions starting from targeted serum dilutions to give 200 ng/ml of IgG, IgA, or IgM, in assay diluent (0.5% BSA//PBS/0.05% Tween-20). Supernatants from stimulated PBMCs were diluted in assay diluent between 1/5 and 1/100. Both standards and samples were allowed to bind for 2 h, washed 6x 300 μl PBST and a 1/5000 cocktail of anti-human kappa-HRP and lambda-HRP antibodies (SouthernBiotech, Birmingham, AL) added for 1 h in assay diluent. After 6x 300 μl PBST washes, the plate was developed with KPL SureBlue™ TMB (VWR).

### BCR Primer Design and Pool Preparation

Dry oligos of desalted purity (IDT) were reconstituted at 100 μM in Qiagen EB and stored in aliquots at −80°C. The oligos are shown in Table S1 and contain sufficient random base pairs to act as unique molecular identifiers (UMIs) for every mRNA transcript present in a sample. UMIs are added in variations of 8 or 12 nucleotide stretches to offset the high level of sequence similarity and lower Illumina sequencing accuracy in Ig amplicons at the 3′ and 5′ ends. A pool of IgH RT primers was made by mixing 10 μl of each primer from the individual 100 μM stocks (100 μl final volume). Separately 10 μl of each of lambda RT primers were mixed from individual 100 μM stocks (20 μl final volume). Next, a 10 μM, 5:1 molar mix of Ig heavy:lambda chain RT primers was made using 16.7 μl IgH RT primer pool and 3.3 μl lambda RT primer pool, in a final 180 μl of Qiagen EB. The same procedure and molar ratio were repeated in the preparation of the IgH (*n* = 12): lambda (*n* = 16) forward primers. Kappa RT and forward primer pools were prepared by mixing 10 μl of each kappa RT (*n* = 2) primer or kappa forward (*n* = 8) primer from the individual 100 μM stocks and then diluting the mix to 10 μM final.

### BCR Amplicon Preparation

Total RNA yields from the PBMC and stimulated PBMCs were in the range of 3 micrograms (50–100 ng/μl in 30 μl Qiagen EB buffer) determined by absorbance at 260 nm on the NanoDrop™ One (Thermo Fisher Scientific). An input of 100–200 ng total RNA was used for first strand cDNA synthesis with gene-specific reverse transcription (RT) primers directed to the constant regions. The RT primers for IgG, IgM, IgA, and lambda were pooled, whereas the kappa RT was done in a separate reaction. Light chain kappa RT was carried out in separate reactions because the transcript abundance and amplification efficiency tended to out-compete heavy and lambda chains in multiplexed reactions. Primers are shown in Table S1. One to two hundred nanograms total RNA was added on ice to 10 μM of pooled RT primers for HC/lambda or kappa chain (primer pools as described above and in Table S1) and 1 mM of dNTP in a 10 μl final volume, allowed to anneal for 3 min at 72°C, and returned to ice. First strand reverse transcription was performed using SuperScript III RT (200 U/μl, Thermo Fisher Scientific). To the 10 μl annealed sample, on ice, 4 μl of 5x Superscript RT buffer, 1 μl 0.1 M dithiothreitol, 1 μl Superase-IN (20 U/μl, Thermo Fisher), and 3 μl of RNase free water were added, to give a final volume of 20 μl. cDNA was made in a thermocycler for 1 h at 50°C, 5 min 85°C, 4°C hold. Second-strand cDNA was synthesized using Phusion High Fidelity Polymerase (Thermo Fisher). To the 20 μl first strand cDNA, 10 μl of 5X Phusion buffer, 1 μl of 10 mM dNTPs, 0.5 μl Phusion Taq, 1.5 μl DMSO, 7 μl of RNase free water, and 10 μl of the 10 μM pool of forward primers (as described above and shown in Table S1), were added, to give a final volume of 50 μl. Samples were incubated at 98°C for 4 min, 52°C for 1 min, 72°C for 5 min and 4°C hold. Double-stranded cDNA was transferred to a low retention DNase-free 1.5 ml Eppendorf tube and purified two times using Agencourt AMPure XP beads (Beckman Coulter, Brea, CA), at a volume ratio of 1:1, and eluted in 25 μl of Qiagen EB buffer. Double-stranded cDNA was PCR amplified with Platinum DNA Polymerase High Fidelity (5 U/μl HiFi Taq, Thermo Fisher). To the 25 μl of eluted 2nd strand cDNA, 5 μl of 10x HiFi Taq buffer, 2 μl of MgSO4, 1 μl 10 mM dNTPs, 0.2 μl HiFi Taq, 1 μl each of two PE primers completing Illumina adapter sequences (Table S1) and 14.8 μl of water, were added, to give a final volume of 50 μl. Samples were run at 94°C for 2 min, 27 cycles of 94°C for 30 s, 65°C for 30 s, and 68°C for 2 min, followed by 68°C for 7 min and 4°C hold. Final libraries were run on 2% E-Gel EX agarose gels (Thermo Fisher Scientific) and bands extracted with Quantum Prep Freeze and Squeeze DNA Extraction Spin Columns (BioRad, Hercules, CA). After one clean-up with 1:1 Agencourt AMPure XP beads, amplicons were eluted in 25–35 μl Qiagen EB. An aliquot was diluted to 5–500 pg/μl and 2 μl quantified on the Agilent Fragment Analyzer Automated CE System using the DNF-474 High Sensitivity, 1–6,000 bp, NGS Fragment Analysis Kit (Advanced Analytical Technologies, Agilent Technologies, Santa Clara, CA), according to the manufacturer's instructions. Pairs of samples (PBMC and stimulated PBMC amplicons) were sequenced together using different Illumina barcodes to demultiplex after sequencing. Amplicon mixtures corresponding to 10:1 ratios of heavy chain + lambda: kappa were submitted for 300 forward × 250 reverse sequencing with MiSeq v3 kits (Illumina) at the Chan Zuckerberg Biohub Genomics Center. Addition of 15–20% PhiX was added to increase sequence diversity and overall sequencing performance. Each MiSeq run resulted in 7.5–20 million paired raw reads, which was reduced to 0.5–3.5 million unique Ig sequences after processing. All MiSeq data is deposited in the SRA database under accession PRJNA524904.

### BCR Repertoire Data Analysis Pipeline

After completion of MiSeq sequencing, antibody repertoires were analyzed using methods based on the Immcantation pipeline (13, 14). An overview of BCR sequencing analysis and practical considerations included in the Immcantation pipeline, are reviewed in Yaari and Kleinstein (15). This pipeline continues to be updated as the field advances, and is composed of multiple software packages: pRESTO, Change-O, SHazaM, TIgGER, and Alakazam, found at https://immcantation.readthedocs.io. Because the Immcantation pipeline can be run using Docker containers, we created a cloud-based workflow incorporating Reflow (https://github.com/grailbio/reflow) that allowed for seamless processing of the constituent Immcantation software packages. The workflow is available at Github (https://github.com/czbiohub/bcell_pipeline). Some key characteristics of our workflow include the use of unique molecular identifiers [UMIs; (16, 17)] at both 5′ and 3′ ends of the Ig sequences, the collapse of sequences with identical UMIs, the use of the IgBLAST algorithm (18) to calculate general Ig characteristics of each sequence, and the determination of clonal families by first calculating a clonal threshold nucleotide distance via a nearest-neighbor algorithm and then collapsing sequences based on this threshold (9). We ran the initial steps using the pRESTO script (presto-abseq.sh at https://bitbucket.org/kleinstein/immcantation/src/97a70949607b6671a182a84d5052b705d1677891/pipelines/?at=default) with variations that are included in our Github repository. Given that our sample and amplicon preparation included UMIs of varying lengths at both 5′ and 3′ ends to improve sequencing quality, we included code to standardize the UMI length for subsequent steps (8 bp at each end). The script first removes reads with average Q scores >20, and then annotates the reads based on 5′ or 3′ amplicon primers. All reads with identical UMIs are then collapsed, with consensus sequences created and UMI numbers annotated into the sequence name. This is followed by assembly of 5′ and 3′ paired-ends, at which point the UMIs at both ends are combined to create a 16 bp signature per cDNA transcript, also annotated into the sequence name. In the next step, the constant regions are re-analyzed for each paired read, and isotype and subtype annotated into the sequence name. The final pRESTO steps include collapsing of identical BCR sequences of the same isotype followed by filtering to only include BCR sequences that were found in 2 or more representative reads per UMI, to avoid including sequences that vary only due to sequencing error. The workflow continues with subsequent Immcantation packages, using the following scripts without changes at the website above: Change-O IgBLAST (changeo-igblast.sh), which calculates Ig repertoire characteristics, TigGER (tigger-genotype.R), which estimates novel V-gene alleles, SHazaM (shazam-threshold.R), which determines the optimal threshold for delineating clonal families, and Change-O Clone (changeo-clone.sh) that groups the sequences into clonal families. Lastly, we use a series of R scripts based on Alakazam that can be found at our Github page to visualize results. This workflow includes both heavy and light chain reads, and all outputs include both sequences, but without knowledge of pairing, therefore we have focused only on the more diverse heavy chain results. In addition to the Immcantation procedure of optimizing the clonal threshold value during each analysis (using SHazaM), we used a second strategy for comparisons across samples to identify mAb matches to clonal families in the repertoire. For these comparisons, we extracted all unique heavy chain sequences (i.e., not only those with 2 or more reads per UMI), appended the mAb sequences, and applied a constant 12% threshold value in Change-O to delineate clonal families. This 12% value was derived using the median of all individual optimized threshold values determined from the multiplexed heavy and light chain data. The Change-O method requires identical V-gene and J-gene usage, CDRH3 length, and in this case up to 12% nucleotide mismatch in the CDRH3 was allowed.

### Preparation of Samples for Bulk Transcriptome Analysis

Double stranded cDNA was prepared according to the single B cell cDNA synthesis described below. cDNA was diluted to range of 0.1–0.3 ng/μl, before tagmentation and PCR amplification with index primers using the Nextera XT DNA SMP Prep Kit and Nextera XT IDX Kit (Illumina, San Diego, CA). After cleanup with Agencourt AMPure XP beads (Beckman Coulter, Brea, CA) the sample was eluted in Qiagen EB buffer and quantified on the Agilent Fragment Analyzer Automated CE System using the DNF-474 High Sensitivity, 1–6,000 bp, NGS Fragment Analysis Kit (Advanced Analytical Technologies, Agilent Technologies, Santa Clara, CA), according to the manufacturer's instructions. Libraries were submitted for 75 × 75 bp sequencing on the Illumina NextSeq High Output instrument. Raw transcriptomic data is deposited in the SRA database under accession PRJNA526542.

### Single Memory B Cell Isolation

Human PBMCs were thawed and stained for 1 h on ice in PBS, 1% BSA (w/v) with antibodies for positive markers of IgG memory B cells, and antibodies to negatively select and avoid contamination with dead cells, T cells, NK cells or monocytes (live, CD3^−^,CD14^−^,CD56^−^, IgM^−^, IgA^−^, CD19^+^,CD20^+^,CD27^+^). Biotinylated HA trimer (A/New Caledonia/20/1999) was complexed using 50 nM at a 4:1 molar ratio with Streptavidin. HA was produced in-house by transiently transfecting Expi293 cells with the extracellular region of HA containing a C-terminal foldon domain for trimerization (19), Avi-tag for biotinylation and 6xHIS tag for Nickel purification using the HisPur™ Ni-NTA Spin Purification Kit (Thermo Fisher Scientific, Waltham, MA). Specific antibody clones and labels are listed in Table S2. The SH800 Cell Sorter was used with 100 μm sorting chips (SONY Biotechnology, San Jose, CA) and NERL™ Diluent 2/Sheath Fluid for Flow Cytometry (Thermo Fisher). Laser compensation was performed using the AbC™ Total Antibody Compensation Bead Kit (Thermo Fisher). Single cells were sorted directly into Hard-Shell® 96-well, low profile, thin wall, skirted, green/clear, PCR plates (BioRad) with 4 μl of lysis buffer, frozen immediately on dry ice and stored at −80°C. cDNA was synthesized according to published Smart-seq2 methods (20). cDNA was cleaned up using 18 μl of Agencourt AMPure XP beads (Beckman Coulter, Brea, CA) in the 25 μl final cDNA volume, washed twice with 170 μl 80% ethanol and eluted in 16 μl of Qiagen EB. Heavy and light chain variable domains were amplified (95°C 5 min, 30 cycles: 95°C 15s, 57°C 30 s, 68°C 1 min; and a final 10 min 68°C extension) from 2 to 3 μl of cDNA in 25 μl of Accuprime Pfx supermix (Invitrogen, Thermo Fisher Scientific) and 0.5 μl of the VH, VK, or VL primer stock mixes described in Table S3. Sanger sequencing primers are also listed in Table S3.

### Recombinant Antibody Cloning, Production, and Purification

Variable heavy or light chain domain PCR products were re-amplified in a nested PCR using primers with at least a 15 base pair overlap matching the 5′ signal sequence and 3′constant region of our human IgG_1_, kappa or lambda expression vectors (see Table S3 for primers). MEDI8852 was made in-house using geneblocks (IDT) corresponding to the published sequence (21). Expression vectors used were in-house constructs of Genbank LT615368.1, deposited by Tiller et al. (22). Assembly of the gene fragments into expression vectors was performed with In-Fusion HD Cloning (Takara Bio USA, Mountain View, CA) and confirmed by Sanger sequencing (Sequetech, Mountain View, CA). Miniprep DNA (5 μg) for the HC and LC of each mAb was transfected into 5 ml of suspension Expi293 cells according to the manufacturer's instructions (Thermo Fisher Scientific) in 50 ml tubes. Cultures were grown in a Multitron shaker (INFORS HT, Annapolis Junction, MD) for 5 to 8 days before supernatants were harvested, clarified, purified and assayed for HA binding. Antibodies were batch purified by adding 500 μl of 1:1 slurry of MabSelect™ SuRe™ (GE, Chicago, IL) overnight. Supernatants and beads were decanted and rinsed into 10 ml Poly-Prep® Chromatography Columns (BioRad, Hercules, CA), washed with 20 volumes of PBS and eluted in 5 ml of 20 mM citrate buffer, pH3, neutralized with 250 μl of 75 mM 1.5 M Tris-HCl, pH8.8. Purified antibodies were concentrated and exchanged by centrifugation into PBS, pH7.2 using Amicon Ultra-15 Centrifugal Filter Units with Ultracel-10 membranes (MilliporeSigma, Burlington, MA). IgG concentrations were determined by OD280 on the Nanodrop One.

### ELISA Measurement of mAb Binding to HA

96-well, Nunc Maxisorp™ plates were coated overnight with 1 μg/ml H1N1 A/New Caledonia/20/1999 hemagglutinin (HA) trimer in PBS, pH7.2. The coated plates were washed 3x 300 μl PBST and blocked for 1 h in 1% BSA/PBS before adding a 1/3 titration of mAbs in assay diluent (0.5% BSA/PBS/0.05% Tween-20) for 1 h at ambient temperature. Plates were washed 6x 300 μl PBST and developed with secondary antibodies and TMB as described above. To measure the binding of mAbs in the presence of MEDI8852 Fab, the MEDI8852 IgG was cut to severe all Fc with LYSYL endopeptidase (Wako Chemicals, Richmond, VA) for 2 h at 37°C. Plates were coated and blocked as described in the binding assay. Each mAb was prepared at a fixed concentration in 0.5% BSA/PBS/0.05% Tween-20 corresponding to a binding OD of 2. A 1/3 dilution series of MEDI8852 Fab was prepared starting from 100 nM. Each mAb was added 1:1 to the Fab dilution series and allowed to bind for 1 h at ambient temperature. Plates were washed 6x 300 μl PBST and developed with an anti-human Fc specific secondary antibody (Invitrogen, Carlsbad, CA) and TMB as described above. Control wells for Fab binding alone were assayed alongside and the final signal subtracted from each corresponding dilution.

## Results

### Experimental Rationale of PBMC BCR Repertoires +/– Stimulation

**Figure 1:**
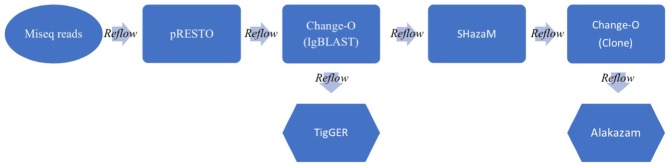
Schematic of the BCR analysis workflow. Raw paired MiSeq reads (300 × 250 bp) are inputted into the Immcantation pipeline. The pRESTO package processes the MiSeq reads and does QC, UMI processing, primer and isotype/subtype annotation and paired read assembly to output annotated sequence lists (.fastq). Change-O next runs the IgBLAST algorithm on all processed Ig sequences, annotating germline information to output tabular files (.tab). TigGER calculates novel germlines, while SHazaM calculates the optimal threshold for clonal clustering. Change-O then clusters the sequences into clones and Alakazam summarizes the outputs in graphical form (.png &.pdf). Throughout the workflow, a Reflow script pieces all of the parts together, delineating outputs and inputs and allowing the pipeline to run in the cloud (using Amazon Web Services).

**Figure 2:**
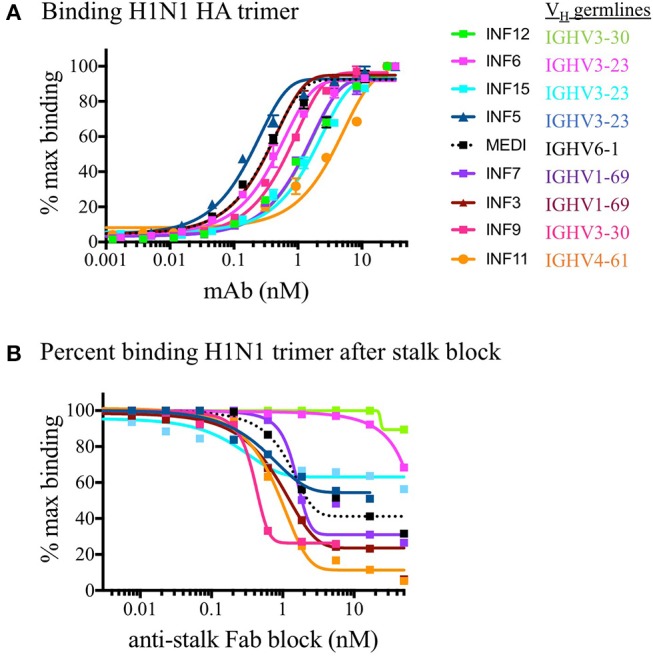
ELISA binding of mAbs to H1N1 HA and functional epitope mapping to the stalk region. **(A)** Purified recombinant monoclonal antibodies were tested for binding to the H1N1 HA trimer compared to the reference in house synthesized MEDI8852 IgG (shown in dotted black line). Replicate data is plotted as a percentage of the maximum in-assay OD_450_ signal which was saturated at 50 nM for all IgG. **(B)** The binding of monoclonal antibodies was measured after blocking the coated HA trimer with increasing concentrations of MEDI8852 Fab. The binding of antibodies INF3, INF7, INF9, and INF11 to the HA trimer were effectively blocked by the Fab fragment of MEDI8852 (Fab blocking the intact MEDI8852 mAb itself is shown in the dotted black line), indicative of common binding of these mAbs to the HA stalk region. In this assay, only INF11 was close to completely blocked at high Fab concentrations. During the 1 h incubation time, higher avidity and affinity IgG (including MEDI8852 IgG) may have reached an equilibrium by displacing bound Fab or by binding to partial epitopes on the trimer independent of the stalk region.

PBMCs from three normal donors of different genetic backgrounds 536 (African American male, 33 yrs), 147 (Caucasian female, 21 yrs.), and 682 (Hispanic male, 30 yrs.) were purified and cryopreserved from Leukapheresis packs. Blood from donor 536 was collected at three different times, in November 2017, May 2018, and August 2018, while donors 147 and 682 were collected once, in August 2018. PBMC vials of 10^7^ cells were processed in one of four ways to obtain (i) PBMC B cell repertoires; (ii) stimulated PBMC B cell repertoires; (iii) bulk transcriptome profiles; or (iv) single, antigen-specific memory B cells. Two vials of cells were processed for each repertoire condition for each of the three donors, in separate experiments, to replicate data. Examples of typical BCR amplicon products used for NGS, which looked similar between PBMC and stimulated PBMC, are shown in Supplementary Image S1. The analysis of Ig repertoires from NGS data is described in detail in the Methods section and a summary of the workflow is provided in Figure 1. Our PBMC stimulation was based on methods previously demonstrated to give rise to the selective expansion, identification, and cloning of antigen-specific memory B cells (12). This method was found to provide the most NGS sequence depth and diversity compared to the alternative approaches of isolating total memory B cells from PBMC by FACS for NGS sequencing or using selective amplification of IgG mRNA from PBMC enriched B cells (Supplementary Image S2). Total IgG, IgM, and IgA secreted by activated B cells was measured between 82 and 954, 29 and 266, and 8 and 35 ng/ml among replicate stimulated cultures of the three donors, respectively (Table 1). These donors' recent infection or vaccination history is not known, but could be reflected in the observed variability of the numbers. For instance, donor 536 may have had a recent exposure at the November timepoint resulting in higher measured IgG and IgM levels relative to other timepoints and donors. The functional *in vitro* stimulation of the PBMCs was further characterized by mRNA expression. Total RNA was used to compare the bulk cellular transcriptome in one replicate of each of the three donors PBMCs before and after stimulation. Upon stimulation, transcripts corresponding to both innate and adaptive immunity were upregulated 10- to 1000-fold over unstimulated PBMCs. Representative genes for T cell and T cell Receptor (TCR) activation, BCR activation and B cell differentiation, as well as genes involved in various innate immune cell activities are shown in Table 2. The stimulation of PBMCs to increase representation of memory B cells should correspond to an improved ability to identify monoclonal antibody sequences in NGS repertoires. However, once a memory B cell is stimulated it loses the transcriptional and cell surface markers of memory and antigen specificity and cannot be identified in a PBMC mixture. Therefore, to test the hypothesis, eight antigen-specific mAbs to the hemagglutinin trimer of influenza H1N1 were obtained by FACS from one donor (536, November blood draw sample), the heavy and light chains sequenced from single cells, and recombinant mAbs made. Their binding activity was confirmed by ELISA using the H1N1 trimer, to which no non-specific IgG binding was observed (data not shown). The mAbs, derived from several different IGVH germlines, demonstrated ELISA binding EC_50_ values in the range of 3–40 nM (Figure 2A), comparable to the high affinity, highly cross-reactive, MEDI8852 mAb directed to the HA stalk region (21). The binding of antibodies INF3, INF7, INF9, and INF11 to the HA trimer demonstrated dose-dependent blocking by the Fab fragment of MEDI8852 (Fab blocking the intact MEDI8852 mAb is shown in black), indicative of common binding to the HA stalk region (Figure 2B). At high Fab concentrations most mAbs reached a plateau level of binding, including the IgG form of MEDI8852. This likely reflects a steady state level of Fab displacement by IgG binding with higher affinity and avidity during the 1 h incubation time. Alternately, some mAbs may be able to retain a low level of equilibrium binding to partial epitopes outside the stalk region. Data identifying these memory B cell mAbs in the repertoires of donor 536 is reported below.

**Table 1:** Immunoglobulin in leukopak supernatant and secreted from stimulated PBMC cultures.

	**Leukopak supernatant μg/mL**					**5-day B cell stimulated media ng/mL**				
	**536N**	**536M**	**536A**	**147A**	**682A**	**536N**	**536M**	**536A**	**147A**	**682A**
IgG	192	129	121	138	190	448	329	278	185	417
(replicate)	–	–	–	–	–	954	374	82	123	362
IgM	483	296	273	212	240	163	96	57	29	57
(replicate)	–	–	–	–	–	266	97	45	34	55
IgA	22	11	14	15	25	24	19	13	8	13
(replicate)	–	–	–	–	–	35	14	11	9	11

*N, November; M, May; A, August*.

**Table 2:** RNAseq TPM for innate and adaptive immune responses observed between PBMCs and *in vitro* stimulated PBMCs^a^^,^^b^.

	**Donor 147**		**Donor 536**		**Donor 682**	
**GENE**	**PBMC**	**Stim-PBMC**	**PBMC**	**Stim-PBMC**	**PBMC**	**Stim-PBMC**
**IMMUNE CELL GENES**						
C1QC	11	1665	3	210	5	275
CCL2	66	11270	41	4201	107	3650
CCR2	25	131	15	94	16	240
CCR5	16	149	1	152	11	100
CD209	22	206	5	51	3	48
CD96	112	759	155	864	99	457
CXCL1	6	1332	3	945	10	327
CXCL8	100	5120	6	5297	2115	679
FCGR1A	5	77	1	17	11	23
GNLY	173	16725	61	34281	1145	15864
GZMB	50	9929	22	23228	138	13583
IL7R	29	620	18	265	106	523
LAG3	11	220	14	259	5	287
LEF1	28	640	20	318	84	336
PRF1	13	2422	16	3739	66	1525
RSAD2	27	130	7	82	6	34
SLA2	27	480	19	511	119	334
SLAMF7	61	629	79	801	92	297
XBP1	750	4768	997	9573	1899	9564
XCL2	67	288	45	592	14	314
ZAP70	125	1891	45	1624	418	1392
KLF2	3845	379	5462	171	3050	467
DUSP1	10458	88	23692	86	16369	84
FOS	4733	73	16289	28	15844	33
BLK	2239	96	1822	45	1499	106
MALAT1	93664	8407	54234	2502	30961	3405
**T CELL GENES**						
CD247	18	302	11	1262	41	241
CD274	10	1180	11	88	112	628
CD3D	44	3023	23	1675	465	2064
CD3E	43	1431	9	1315	322	931
CD3G	10	1979	15	269	33	1496
CD7	24	3960	10	723	120	1723
CD8A	14	435	9	1337	132	463
CD8B	14	531	20	1674	87	576
CISH	37	688	23	2026	120	443
EOMES	25	886	9	305	139	536
HAVCR2	74	1271	12	760	93	917
LAT	33	761	27	1725	62	417
PRKCQ	46	365	18	350	44	250
SIT1	52	224	44	523	17	207
SPN	12	91	9	421	13	72
TRAT1	46	513	2	280	35	244
TXK	13	191	15	208	16	214
ZNF683	60	407	11	211	27	159
CD69	2785	472	4815	336	3263	319
**B CELL GENES**						
CD27	145	1199	154	1363	93	1249
CD38	14	141	23	218	4	117
IGLL5	2465	8230	2102	21659	896	25995
JCHAIN	461	1945	737	7780	252	9099
PRDM1	37	198	47	383	67	254
UNG	21	250	8	260	6	191
CD22	941	101	1854	99	572	136
CR2	33	4	30	7	11	3
CD79A	14254	1520	12293	1980	9219	4541
LYN	923	185	1290	125	780	153

a *August timepoint; Most highly upregulated genes from Adaptive Immune Response GO_0002250*.

b *TPM = reads (mapped to only one gene) normalized for gene length and then normalized for sequencing depth*.

### Equivalent Germline Usage in PBMC and Stimulated PBMC Repertoires

**Figure 3:**
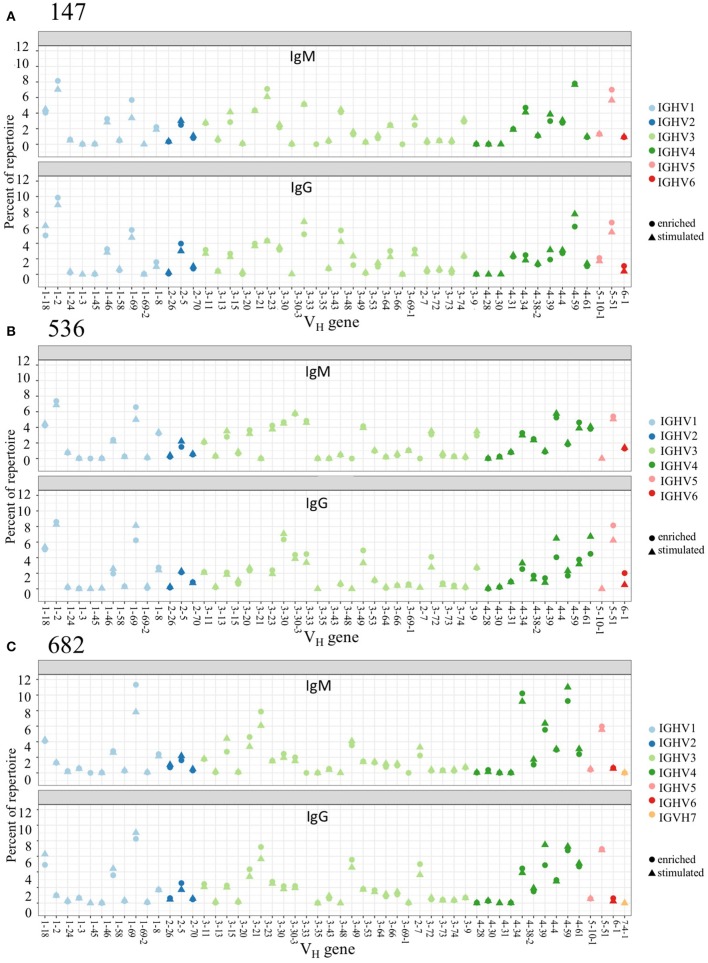
Germline V-gene usage among PBMC and Stimulated PBMC repertoires. Plots show V-gene usage for one replicate of each of the three donor repertoires (**A**: 147, **B**: 536, and **C**: 682) sampled at the August 2018 timepoint, and are separated by isotype, with only IgM and IgG sequences shown. Plots were made using the Alakazam R scripts in the Immcantation pipeline. Corresponding V-gene usage plots for light chains are in Supplementary Image S3.

We examined PBMC and stimulated PBMC repertoires using our workflow based on the Immcantation pipeline, using an optimized clonal threshold value for each sample and limiting data to sequences with at least 2 representative reads per UMI. Comparing V-gene usage between replicate repertoires, we found that in both PBMC and stimulated PBMC repertoires gene usage was consistent with previously described healthy BCR repertoire data (23), and germline usage was equivalent across PBMC and stimulated PBMC repertoires (e.g., VH in Figure 3, and VK/VL in Supplementary Image S3). For example, examining IgG and IgM sequences in one replicate at one timepoint, VH1-2, VH3-23, VH3-33, VH4-59, and VH5-51 are among the most commonly used V-genes across both PBMC and stimulated PBMC repertoires in donor 147, VH1-2, VH1-69, VH3-30, VH4-39, and VH5-51 are among the most commonly used V-genes across both PBMC and stimulated PBMC repertoires in donor 536, and VH1-69, VH3-23, VH4-39, VH4-59, and VH5-51 the most commonly used V-genes across both PBMC and stimulated PBMC repertoires in donor 682. The largest differences in gene usage between PBMC and stimulated PBMC repertoires occur in the most commonly used antigen-specific V-genes, particularly VH1-69, VH4-39, and VH4-59.

### Stimulated PBMC Repertoires Shift to More Antigen-Experienced Sequences

**Figure 4:**
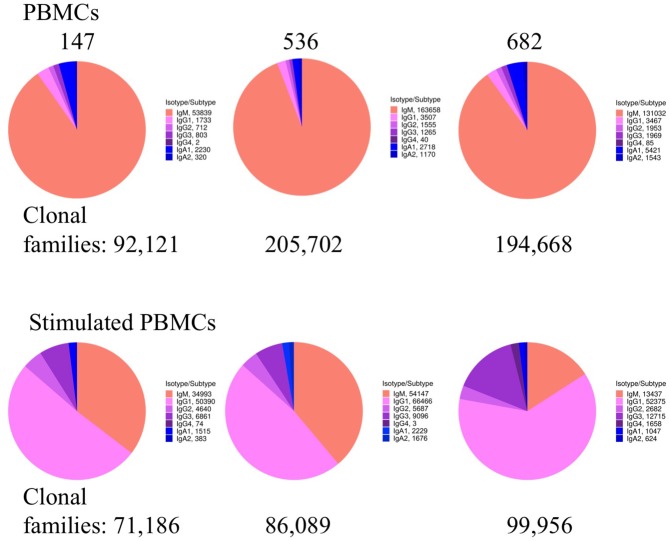
Heavy chain isotype and subtype usage among PBMC and stimulated PBMC repertoires. Plots show the proportion of heavy chain isotypes for one replicate of each of the three donors (147, 536, and 682) sampled at the August 2018 timepoint. While pie chart numbers show unique Ig sequences found with >2 reads per UMI, the more stringent criteria for enumerating clonal families by V-D-J sequence similarity is also shown (in bold numbers on pie charts). A constant clonal threshold value of 12% was used to assign sequences into clonal families, so data could be compared across samples (see Methods). Plots were made using the Alakazam R scripts in the Immcantation pipeline.

**Figure 5:**
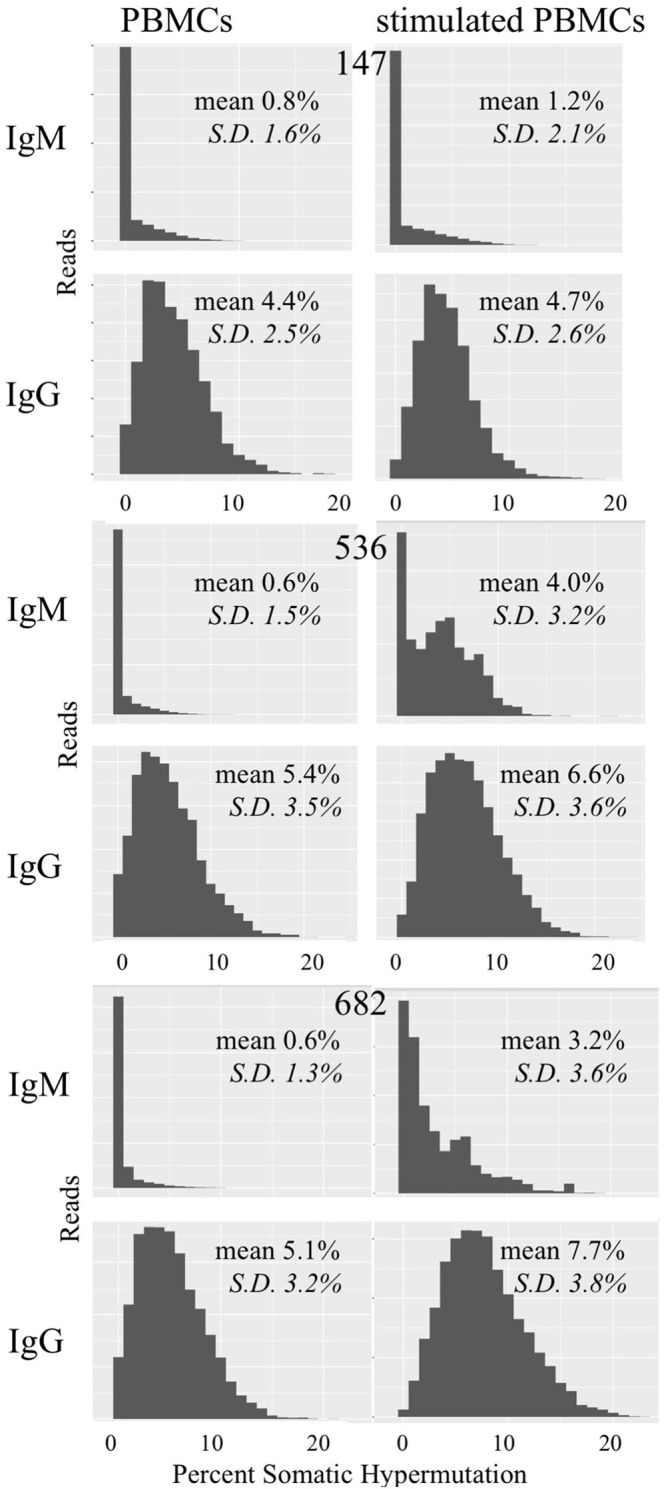
Somatic hypermutation among PBMC and stimulated PBMC repertoires. Plots show distributions of SHM values across all IgM and IgG sequences in PBMC repertoires vs. stimulated PBMC repertoires in one replicate, for each of the three donors (147, 536, and 682) sampled at the August 2018 timepoint. Plots were made using the Alakazam R scripts in the Immcantation pipeline. Since the total number of sequences in each distribution plot are different, to compare across samples, the y-axis were normalized to the maximum number of sequences.

We examined whether PBMC and stimulated PBMC repertoires vary in their antigen experience by quantifying heavy chain isotype ratios and SHM among IgM and IgG sequences. To more accurately calculate SHM, we used the Immcantation package TigGER which includes inferred novel alleles in downstream SHM calculations. TigGER found between 3 and 11 novel alleles per donor (refer to Table S4). Among replicate samples of three donors, we found a consistent shift in repertoires with respect to the proportions of heavy chain isotype and subtype content. In PBMC repertoires, IgM made up over 90% of all heavy chain sequences across all samples and replicates. In contrast, stimulated PBMCs are greatly enriched for IgG and particularly the IgG_1_ subtype, comprising between 58 and 82% of all heavy chain sequences (Figure 4, Table 3). Somatic hypermutation frequencies (Figure 5) also significantly increased in the 3 different donors, although to varying degrees (Student's *t*-tests with Holm-Sidak correction, yielded *p* < 0.001 values for all comparisons). Variations in SHM may reflect the donors exposure history and immune status at the time of the blood draw. PBMC repertoires averaged 0.7% SHM in IgM sequences and 5.1% SHM in IgG sequences (Table 3A), while stimulated PBMC repertoires averaged 2.2% SHM in IgM sequences and 6.0% SHM in IgG sequences (Table 3B).

**Table 3A:** Summary of Ig sequences^a^, clonal families, and their corresponding mean SHM in two experimental replicates of PBMCs.

**PBMCs^b^**	**147A**		**536N**		**536M**		**536A**		**682A**	
HC sequences	90398	59651	112128	144519	139389	186664	253586	173944	239491	145578
HC clonal families	75672	49595	95095	102093	119668	148994	204129	145749	202146	129432
IgM sequences	82607	53839	103577	132636	129498	174385	238019	163658	217268	131032
SHM, IgM sequences	0.8%	0.8%	0.8%	1.0%	0.8%	0.8%	0.6%	0.6%	0.6%	0.5%
IgG sequences	4768	3255	4760	7174	5481	7135	8735	6389	12916	7566
SHM, IgG sequences	4.4%	4.3%	5.5%	5.4%	5.5%	5.4%	5.4%	5.4%	5.1%	5.1%
IgA sequences	3023	2557	3791	4709	4410	5144	6825	3897	9307	6980
SHM, IgA sequences	4.6%	4.6%	4.8%	4.9%	4.9%	4.8%	4.9%	4.8%	5.6%	5.4%
Unique clonal family threshold^c^	12.8%	15.0%	9.7%	18.0%	9.2%	10.9%	8.5%	8.2%	6.0%	8.0%
IgM clonal families	68789	44406	87385	92265	110789	138560	190087	136855	183286	117115
SHM, IgM clonal families	0.6%	0.6%	0.6%	0.6%	0.6%	0.5%	0.4%	0.4%	0.5%	0.5%
IgG clonal families	4231	2935	4277	5815	4900	6000	7286	5427	11505	6637
SHM, IgG clonal families	4.3%	4.2%	5.3%	5.1%	5.5%	5.2%	5.3%	5.3%	5.1%	5.0%
IgA clonal families	2652	2254	3433	4013	3979	4434	5798	3467	7355	5680
SHM, IgA clonal families	4.5%	4.5%	4.7%	4.6%	4.8%	4.7%	4.8%	4.8%	5.4%	5.4%
HC all unique reads	512326	305131	518555	978166	641993	1036751	1116641	965622	1167074	836912
IgM clonal families, 12%^d^	123144	84103	155977	191438	160517	212548	275685	191826	278478	178061
IgG clonal families, 12%	6943	4458	7411	11093	6926	10129	11354	8279	14739	9021
IgA clonal families, 12%	4052	3560	6143	7719	5861	7724	9392	5597	8524	7586

a *sequences with reads per UMI ≥ 2*.

b *N, November 2017; M, May 2018; A, August 2018*.

c *redundant and variable amounts of amplified light chains multiplexed with the heavy chains for sequencing contributed to variability in these numbers*.

d *12% = secondary clonal threshold based on 12% distance, using all unique reads*.

**Table 3B:** Summary of Ig sequences^a^, clonal families and their corresponding mean SHM in two experimental replicates of *in vitro* stimulated PBMCs.

**Stimulated PBMCs^b^**	**147A**		**536N**		**536M**		**536A**		**682A**	
HC sequences	128312	99898	71594	124013	185986	179813	51720	142435	58893	89055
HC clonal families	72805	41404	12626	19424	64264	86379	15838	51756	21798	26944
IgM sequences	69335	34993	13295	28893	64401	85831	6039	54147	2430	13437
SHM, IgM sequences	1.2%	1.1%	4.2%	2.8%	1.8%	1.3%	4.0%	1.2%	3.4%	1.4%
IgG sequences	55161	62984	55233	89078	113665	87748	44577	84324	56846	73874
SHM, IgG sequences	4.7%	4.5%	6.4%	6.5%	6.3%	6.1%	6.6%	6.7%	5.8%	6.2%
IgA sequences	3816	1921	3066	6042	7890	6234	1104	3964	606	1744
SHM, IgA sequences	4.9%	4.7%	6.1%	5.9%	5.8%	5.7%	5.9%	5.8%	6.3%	6.3%
Unique clonal family threshold^c^	13.6%	12.0%	19.0%	18.0%	8.4%	11.5%	19.3%	8.6%	20.2%	12.9%
IgM clonal families	52584	27928	3267	12249	42532	63103	2034	38535	1258	10068
SHM, IgM clonal families	0.7%	0.7%	1.5%	0.8%	0.7%	0.6%	1.9%	0.5%	2.1%	0.7%
IgG clonal families	17331	12179	8557	5896	18706	19822	13321	11631	20438	15465
SHM, IgG clonal families	4.6%	4.6%	6.0%	6.1%	6.2%	6.1%	6.0%	6.5%	5.5%	5.8%
IgA clonal families	2620	1297	802	1279	3026	3454	483	1590	377	791
SHM, IgA clonal families	4.7%	4.8%	6.1%	5.6%	5.5%	5.4%	6.2%	5.6%	6.1%	6.0%
HC all unique reads	1071506	689021	768278	875935	1564721	953708	627655	971885	1103074	1046912
IgM clonal families, 12%^d^	78685	39000	23783	19200	36281	80939	17171	54315	19747	54699
IgG clonal families, 12%	41078	30372	26472	18338	46555	39857	33954	28099	65559	42795
IgA clonal families, 12%	3523	1814	3792	3472	8451	6632	1299	3675	1541	2462

a *sequences with reads per UMI ≥ 2*.

b *N, November 2017; M, May 2018; A, August 2018*.

c *redundant and variable amounts of amplified light chains multiplexed with the heavy chains for sequencing contributed to variability in these numbers*.

d *12% = secondary clonal threshold based on 12% distance, using all unique reads*.

### Increased Sensitivity for Detection of Antigen-Specific mAbs From Stimulated PBMC Repertoires

**Figure 6:**
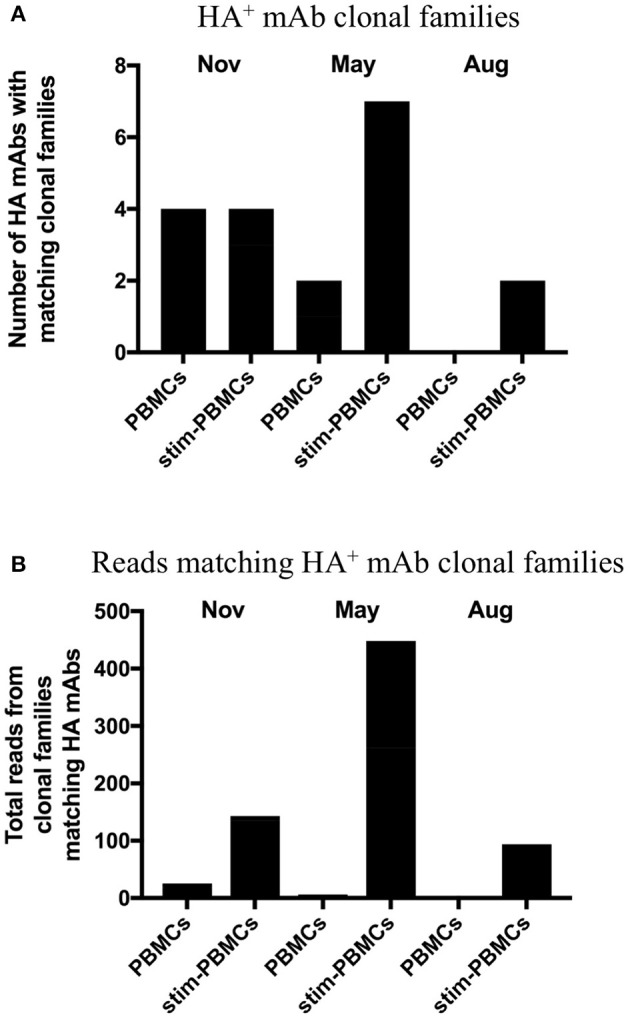
Clonal families of 8 HA^+^ memory B cell sequences found among PBMC and stimulated PBMC repertoires. **(A)** Number of mAbs out of the eight HA^+^ memory B cell sequences found with clonal families in either PBMC repertoires or stimulated PBMC repertoires, for all three timepoints of donor 536. One mAb (INF15) was not found in any of the repertoires. **(B)** Number of sequences found within these clonal families, in either PBMC repertoires or stimulated PBMC repertoires for all three timepoints of donor 536.

In order to identify and quantitatively compare single cell mAb matches to clonal families among different repertoire samples from a given donor, we included all unique heavy chain sequences, appended the focal mAb sequences, and applied a constant 12% clonal threshold nucleotide distance during analysis. By using a constant clonal threshold value to apply across sample replicates and timepoints rather than allowing the threshold to be a variable, we thus provide a consistent delineation between sequence relationships in the datasets being compared. In our searches for single cell memory mAbs in donor 536, we found matching clonal families for the influenza mAbs most frequently among stimulated PBMC repertoires across all blood draw timepoints (Figure 6A). These hit rates improved despite the fact that the total number of clonal families in stimulated PBMC repertoires were up to 12-fold lower than PBMC (e.g., 204,129 vs. 15,838 in a single August replicate for donor 536; Tables 3A,B), because sequences were enriched for the IgG switched and higher SHM phenotype of antigen-experienced BCR (Figures 4, 5). As would be expected from analyzing samples from a dynamic immune system, we observed differences in mAb matches to Ig repertoires among the timepoints. As shown in Figure 6A, mAb searches at the initial timepoint (November), when the mAb B cells were isolated, showed equal hits for PBMC and stimulated PBMC (4/8 mAbs). The second timepoint (May) had the greatest number of matches in the stimulated PBMC repertoire (7/8 mAbs), relative to the PBMC (2/8), while the number at the third timepoint (August) dropped to 0/8 mAbs in PBMC and 2/8 mAbs in stimulated PBMC. The donor was collected as healthy and no medical history is known, but these findings could be consistent with this donor having received exposure or vaccination to influenza A virus just prior to the November blood draw, reducing the sampled diversity during selective clonal expansion, with the immune response waning in the PBMC compartment by the following August. It is also possible that this donor experienced additional exposures to Influenza A between the November and May blood draws, further reinforcing memory clones. In addition to identifying mAb clonal families in the stimulated PBMC repertoires more frequently, we also found considerably more sequences matching these clonal families (Figure 6B). Out of 5 million total processed Ig sequences with unique UMI across PBMC repertoires for November, May and August, sequences matching the mAb clonal families ranged from 0 to 18, while in stimulated PBMC repertoires, matching mAb sequences ranged from 95 to 448 out of a total of 7 million processed Ig sequences. Activated B cells proliferate and significantly upregulate mRNA transcription, thus making clonal families of interest more easily found, and found in larger numbers. Further analyses of the relative size and diversity of the mAb clonal families at the May timepoint, are described in sections below.

### Persistent BCR Sequences With Antigen Experience Are Found More Frequently in Stimulated PBMC Repertoires

**Figure 7:**
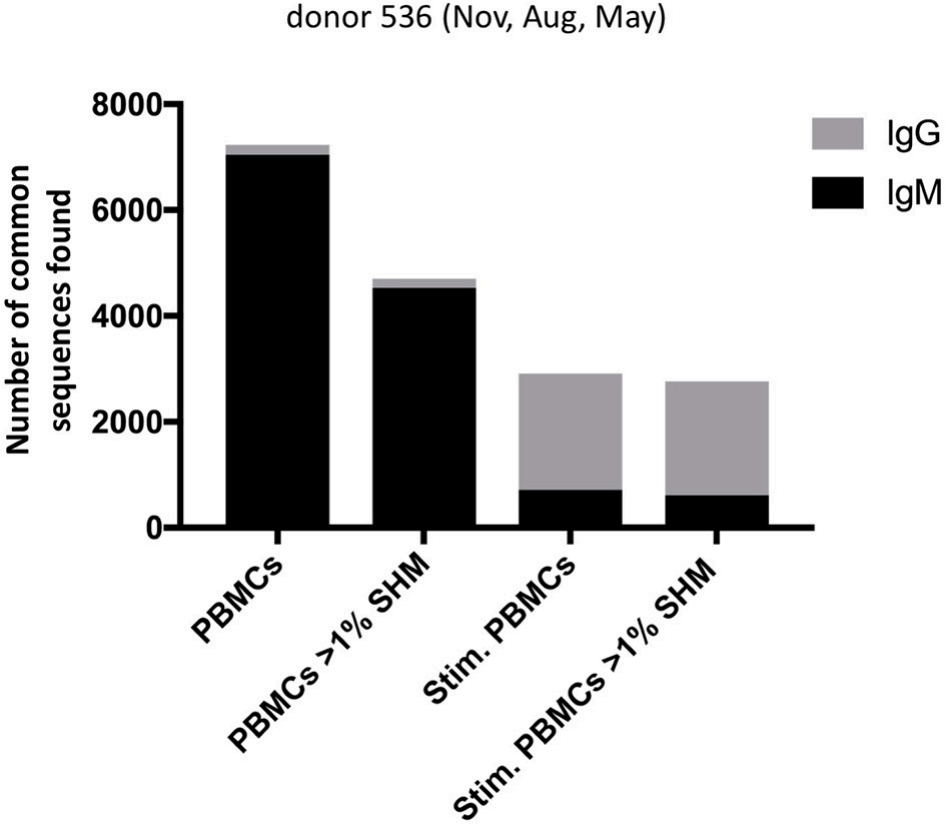
Number of persistent sequences found in all three timepoints in a single donor, among PBMC or stimulated PBMC repertoires. Each column indicates the number of identical heavy chain sequences for IgM (black) or IgG (gray) sequences found across all three timepoints of donor 536, for PBMC or stimulated PBMC. Either total sequences or mutated sequences (>1% SHM) are shown as indicated on the x-axis.

With the three samples of PBMCs drawn from a single donor, we examined whether there were “persistent” BCR sequences found across all timepoints spanning a 9 month period, continuing with the strategy including all unique heavy chain sequences and applying a constant 12% clonal threshold. Identical BCR sequences found consistently over time would more likely be derived from the memory B cell population, particularly if they have switched to the IgG isotype and have V-D-J sequences mutated from germline. Searches for identical BCR sequences were conducted in Geneious v11.1 (Biomatters, Auckland, New Zealand) on data merged from the replicate experiments, using DNA segments corresponding to FR1 residue 12 to FR4 residue 136 (Kabat numbering). Among 5 million processed Ig sequences from donor 536 PBMC across November, May and August, we found 7229 BCR sequences identical at the nucleotide level that persisted across all timepoints, of which only a small fraction (173) were mutated IgG BCR sequences. In contrast, out of 7 million corresponding donor 536 stimulated PBMC sequences, 2,908 identical BCR sequences persisted across the three timepoints, of which the majority (2,151), were mutated IgG BCR sequences (Figure 7). The BCR sequences had unique UMI usage between datasets, making it unlikely they were the result of cross-contamination. UMI codes are in the primers at the RT step where they are uniquely incorporated into a single cDNA. Also, the subsequent PCR reaction adds index primers to the UMI to differentiate reads between samples, further preventing incorrect sequence assignments downstream. It would be expected that, over this individual's lifetime, the sequences of many of these persistent clones, observed over a 9-month period, would change. Longitudinal studies would be useful to evaluate lifetime immune repertoires circulating in PBMC, and how these relate to Ig sequences in serum (24).

### Application of Stimulated PBMC Repertoire Data to Determine the Immunological Hierarchy of B Cell Clonal Memory

**Figure 8:**
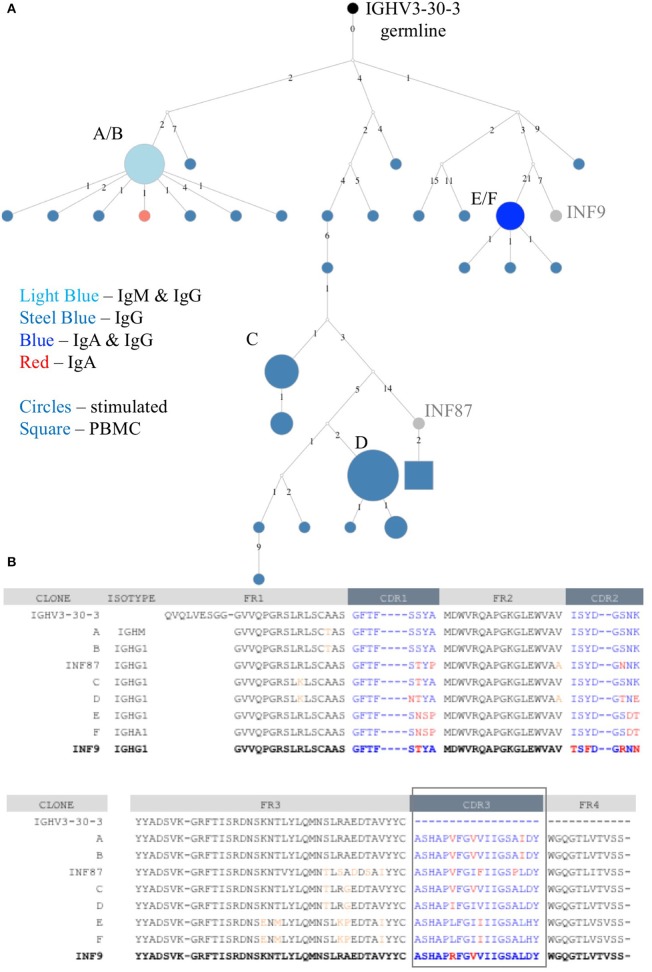
Clonal family lineage of Influenza A HA^+^ memory B cell mAb INF9. **(A)** The clonal lineage of mAb INF9 deriving from the germline (IGHV3-30-3) in black, with IgG sequences in steel blue, IgM/IgG sequences (i.e., identical except for constant region) in light blue, IgG/IgA sequences in royal blue, one IgA sequence in red, and reference mAb B cell sequence INF9 shown in gray. White dots indicate putative sequences not found. INF87, found toward the bottom of the lineage and shown in gray, is another memory B cell sequence cloned after HA^+^ FACS but not tested to confirm HA-binding. The sizes indicate the relative read count per UMI. The sequences in this lineage were pooled from two replicates each of the PBMC and stimulated PBMC repertoires from the May 2018 timepoint of donor 536. Only a single sequence, shown as a square at the bottom of the lineage was derived from the unstimulated PBMC. This sequence was different by two nucleotides but identical at the amino acid level to INF87. The lineage was made using the Alakazam R scripts in the Immcantation pipeline, with a parsimony-based approach. Numbers between sequences indicate mutational steps at the nucleotide level. **(B)** An amino acid alignment of variable heavy chain sequences selected from the clonal family lineage shown in A. Somatic hypermutation from germline in the CDR1, CDR2, and CDR3 regions are shown in red and in the framework (FR) regions in orange. The reference mAb INF9 is shown on the bottom line, in bold.

**Figure 9:**
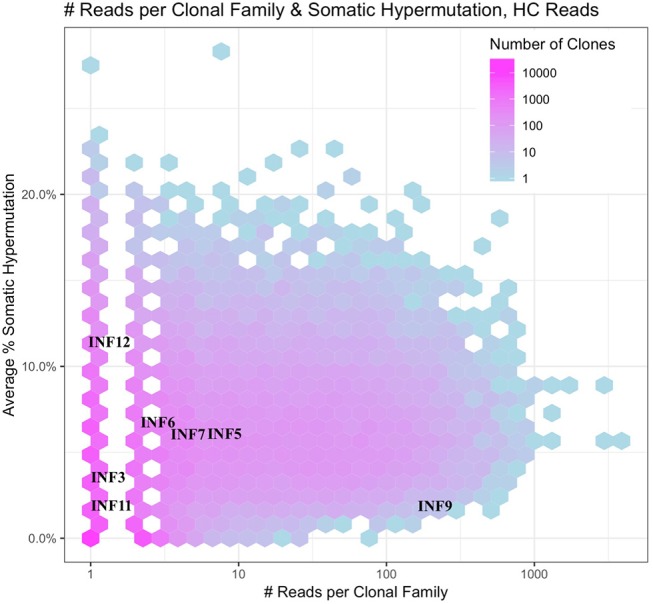
Immunodominance plot of the May stimulated PBMC repertoire of donor 536. Clonal families of mAb IGVH sequences were found and plotted onto a heat map made from the total May IGVH clonal repertoire. The number of unique clonal families in the May IGVH repertoire at a given abundance and average SHM is indicated using a color scale, ranging from pinks for >1,000 unique clonal families to blues for <10 unique clonal families. Since the heat map plots all clonal families from the repertoire at a given abundance and average SHM, individual mAb clonal family diversity cannot be interpreted by color. The y-axis shows the average somatic hypermutation of clonal families and the x-axis the abundance of clonal families by number of sequencing reads. Because whole numbers are plotted using a log scale on the x-axis, this gives rise to an appearance of gaps between the lowest values. The plot was made using the Alakazam R scripts in the Immcantation pipeline.

The deeper memory BCR repertoire data obtained from stimulated PBMCs allows for the identification of large clonal families related to known antigen-specific mAbs, making it possible to examine the expansion of antigen-specific mAbs in detail. For instance, we were able to map HA-specific mAbs onto lineages or networks of clonal families, highlighting aspects such as isotype, subtype and abundance (by reads per UMI). For example, the clonal lineage of HA stalk-region binding antibody INF9 is shown in Figure 8. The representation was made using the Alakazam package in the Immcantation pipeline and is a parsimony-based network of the sequences clonally related to INF9 (in donor 536, single replicate of May timepoint), using data that could only be found in the stimulated PBMC repertoire, with the exception of a single sequence from the unstimulated PBMC repertoire, found near the bottom of the lineage. Within the lineage, INF9 itself appears as a minor member, with other BCR sequences having been selected for expansion and SHM divergence. Although IgM and IgA isotypes were observed, IgG dominated the response. We can also examine the relative abundances of antigen-specific mAb clonal families in stimulated PBMC repertoires. For example, using the stimulated May repertoire of donor 536, containing 1 million processed Ig sequences and a total of 127,448 clonal families (at the 12% clonal threshold), we created a heat map visualization of all clonal families (Figure 9) plotted with SHM on the y-axis and the abundance (reads per clonal family) on the x-axis. The color indicates the number of clonal families in the dataset with a particular SHM/abundance characteristic from high (bright pink) to low (light blue). After finding heavy chain clonal families for 7/8 mAbs, we mapped their values for average SHM and abundance onto the heat map (Figure 9). In this example, mAb INF9 has the most abundantly expressed clonal family of the 8 antigen-specific mAbs, containing 166 different sequences, and a 3% average SHM. In comparison, mAb INF12 shows the highest average SHM (12%) and low abundance (1 sequence) in the May repertoire. Despite being of low abundance in the May stimulated PBMC repertoire, sequences of variable read counts per UMI related to INF12 were found across all timepoints, indicating its persistence (data not shown). With more sample data and extensive mAb mapping, it may be possible to assess the relative immunodominant hierarchy of B cell clonal memory *in vivo*, which could be correlated to past and future protection from disease and guide vaccine design.

## Discussion

There is an ongoing need for the development of improved vaccines to replace those that have high production costs, poor stability, efficacy, and/or durability as well as a need for research to inform new vaccines for intractable or emerging pathogens. Even for the most successful vaccines, continued vaccination of global populations and surveillance is often necessary to offset the re-emergence of diseases after incomplete geographical or lifespan coverage of immune protection. Furthermore, the natural evolution of pathogens to new or vaccine resistant strains provides an ongoing challenge to protective strategies. An ideal vaccine would provide robust, directed immunogenicity to give rise to dominant clones of high affinity antibodies with specificity, cross-reactivity and pathogen neutralizing or clearing activity. Importantly, clonal families for the corresponding antibody producing B cells would be found to be consistently well-represented in the memory compartment, with the ability to be rapidly and functionally activated upon subsequent exposures.

In this paper, we provide methods to allow for a deep analysis of the clonal diversity, persistence and hierarchy of antibodies in the memory repertoire from PBMCs. Our approach paralleled that of ELISPOT methods used to track vaccinations, whereby memory B cells are polyclonally differentiated into antibody secreting cells and expanded within the autologous PBMC environment. By comparing PBMC repertoire data in three healthy donors before and after stimulation, we showed the corresponding memory B cell Ig mRNA was better represented in amplicon pools of PBMC after stimulation, which are otherwise dominated by naïve, less antigen experienced B cell sequences. Definitive memory phenotype characteristics are lost upon B cell activation and we were not able to characterize single memory B cells from stimulated PBMCs. Instead, to support that the repertoire sequences we enriched for were from memory cells, we cloned single, antigen-specific memory B cells by standard cell surface FACS markers and demonstrated the mAb clonal family sequences could be more readily identified and found at greater depth in the stimulated PBMC repertoires. Furthermore, we were able to use the richer clonal family data to rank the immunodominance of mAbs. Such ranking could provide an informative tool to quantify the functional quality and direction of an immune response, such as in our examples, toward known neutralizing epitopes on the Influenza A hemagglutinin stalk region.

The correlation between the *in vitro* stimulation methods used here and functional *in vivo* memory B cell lineages is not known. Understanding how antigen-dependent and independent signals co-operate *in vivo* to maintain, activate and eliminate B cell populations is challenged by the complex and contextual environment of immune responses and regulation. Nevertheless, several lines of evidence in our data support that *in vitro* stimulation is not simply expanding clonal families by random SHM: (i) among identical V-D-J sequences (at the nucleotide level) found in repertoires of the same donor over time, a higher fraction were mutated IgG in stimulated PBMCs (74%) vs. PBMCs (2%); (ii) identical and near identical V-D-J sequences are found matching memory B cell, antigen-specific monoclonal antibodies isolated by FACS from the same individuals PBMC; (iii) sequences matching those in (ii) are found in repertoires of different blood draws taken over time from a given donor; (iv) sequences matching those in (ii) are found with a 10-fold or greater read number in stimulated PBMC which would correlate with increased cell proliferation and/or mRNA transcription in the absence of SHM; (v) sequences from stimulated PBMC are frequently distributed in clonal lineages closer to the germline, with fewer mutations than the reference mAbs; and (vi) highly cross-reactive viral-binding mAbs isolated during the acute phase of a secondary infection map to large clonal expansions and to distal points of clonal family lineages in acute phase stimulated PBMCs from within the same patient and, with lower frequency, among different patients (manuscript in preparation). To better understand *in vitro* stimulated lineages we are currently making and testing the activity of a variety of VH mutations obtained by NGS in these acute phase samples, paired with the reference mAb light chain. Notably, calculations using the Immcantation pipeline on our stimulated PBMC NGS data also provide a means to estimate mAbs in the context of the overall number of clonal families attributed to human memory B cells in blood. We observed in the range of six to twenty thousand clonal families for IgG memory among the three donors, while previous work has estimated the plasma cell derived clonal repertoire size in serum to be on the order of 20,000 (24). Additional data analysis is warranted to capture the variability between replicates and a statistical saturation point. Applying the new Immcantation tool called Repertoire Dissimilarity Index [RDI; (25)], we determined RDI scores based on heavy chain V-gene usage (without D and J) between our replicates of 2.26 in PBMC repertoires and 2.36 in stimulated PBMC repertoires, suggesting the repertoires were not fully sampled. The methods for how threshold values are determined and applied to calculate and compare clonal families is an area warranting further study, and *in vitro* expansions of clonal families will offer deeper data sets for testing. In future studies, it will be of interest to compare the repertoire of polyclonal *in vitro* stimulation of B cell memory with larger sets of antigen-specific mAbs, and to use that data to identify public (many donor) vs. private (individual donor) sequences. Overall, the analysis of deep functional memory repertoires *in vitro* will provide for a better view of the *in vivo* landscape of humoral protection or enhancement of disease, informative to vaccine, autoimmune and therapeutic antibody research.

Clonal expansions of B cells occur in response to specific immune stimulation and evidence for a corresponding convergence of germline gene arrangements has been shown [e.g., (23–27)]. Clonal families represent *in vivo* antibody molecular evolution to a consensus directed against common epitopes of vaccines or disease. As the field gains more and more knowledge on immune repertoires and collects data on responses to disease and vaccination, we will get closer to being able to deliberately direct our immune system toward epitopes yielding positive health outcomes.

## Data Availability

The datasets generated for this study can be found in the NCBI Sequence Read Archive (SRA) under accession PRJNA524904 (MiSeq data) and PRJNA526542 (transcriptomic data) at https://www.ncbi.nlm.nih.gov/sra/?term=PRJNA524904 and https://www.ncbi.nlm.nih.gov/sra/?term=PRJNA526542.

## Author Contributions

KM conceived and designed the study. EW, NF, and KM carried out the experiments. EW and KM performed data analysis and wrote the manuscript. EW and AM developed the sequence analysis pipeline. PK provided study oversight and review. All the authors read and approved the final manuscript.

### Conflict of Interest Statement

The authors declare that the research was conducted in the absence of any commercial or financial relationships that could be construed as a potential conflict of interest.
