# Supplementary material for: Functional Enrichment and Analysis of Antigen-Specific Memory B Cell Antibody Repertoires in PBMCs
Source: Front Immunol. 2019 Jun 25;10:1452. doi: 10.3389/fimmu.2019.01452 (PMC6603168; doi:10.3389/fimmu.2019.01452)

SUPPLEMENTARY IMAGE S1. Amplicon agarose gel purification and fragment analyzer quantitation.

(A) PCR amplicons of the human immunoglobulin heavy/lambda light chain and kappa light chain from total cellular RNA are shown on a 2% agarose gel. The white boxes indicate the regions excised from the gel for extraction.

(B) Examples of fragment analyzer results for the final extracted and purified amplicons immediately prior to sequencing submission are shown with the V_H at 622bp, V_{lambda} 532bp (upper plot) and V_{kappa} at 556bp (lower plot). Peaks labeled LM and HM are low and high molecular size markers, respectively. Areas under the curves are used for quantitation, with typical yields being in the range of 5ng/µl in 25-35µl.

(A)

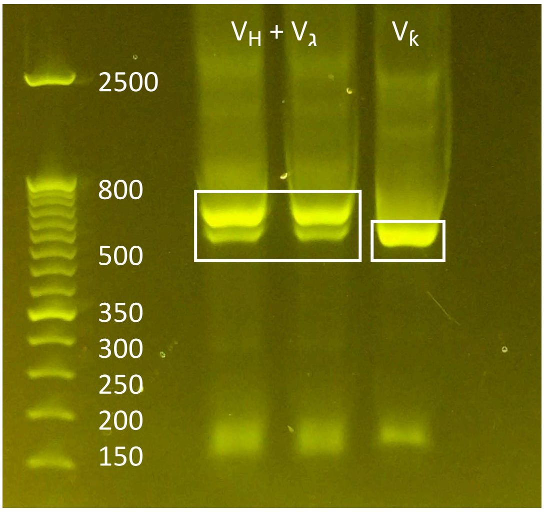

(B)

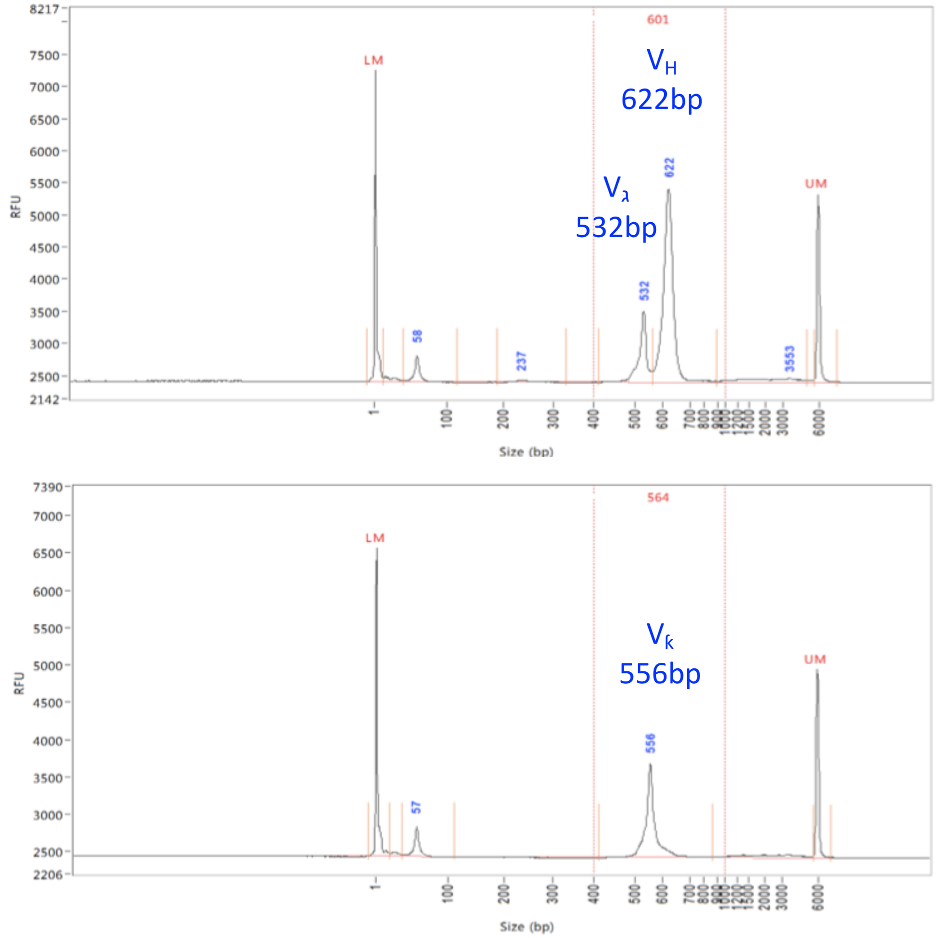

Supplement: Supplementary file 2 [file Data_Sheet_1.PDF]
