# Supplementary material for: Functional Enrichment and Analysis of Antigen-Specific Memory B Cell Antibody Repertoires in PBMCs
Source: Front Immunol. 2019 Jun 25;10:1452. doi: 10.3389/fimmu.2019.01452 (PMC6603168; doi:10.3389/fimmu.2019.01452)

SUPPLEMENTARY IMAGE S2. A comparison of memory Ig repertoire enrichment methods.

Plots show heavy chain V-gene proportions for one replicate of donor 536 sampled at the November 2017 timepoint (A, B, C) and at the May 2018 timepoint (D, E, F). Each of the repertoires used 10 million PBMC processed in different ways as (see Methods): A, D and F were enriched by magnetic selection for total B cells, B and E were stimulated *in vitro* as PBMC, and C was sorted by FACS for memory B cell markers (yield was 100,000 cells). Total RNA was amplified with all Ig reverse primers except in sample F where only the IgG reverse primers were used. Stimulation of PBMC provided the largest and most diverse sampling of the IgG repertoire (B, E). Pie chart numbers show unique Ig sequences found with >2 UMIs, with IgG reads also shown in bold. Plots were made using modifications of the Alakazam R scripts in the Immcantation pipeline.

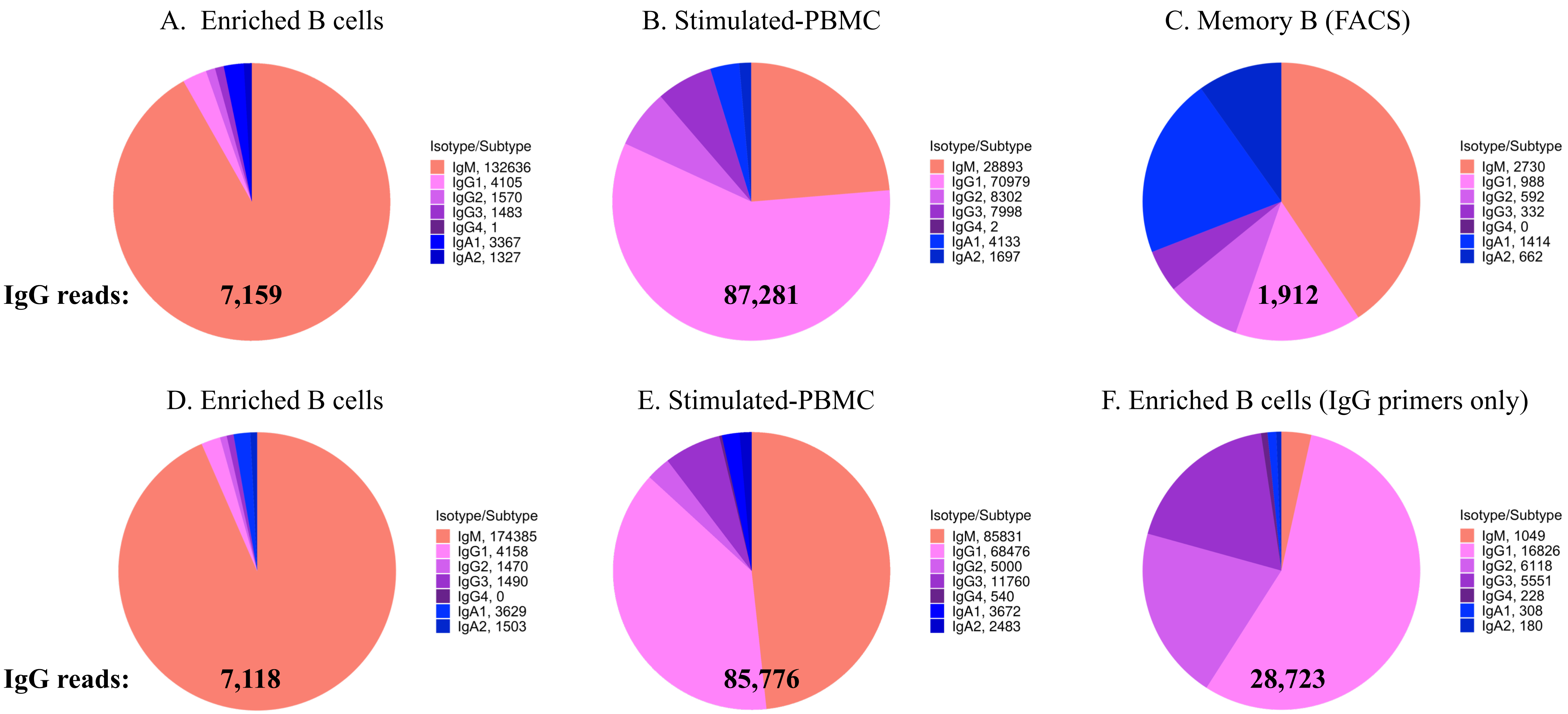

Supplement: Supplementary file 3 [file Data_Sheet_2.PDF]
