# Supplementary material for: Functional Enrichment and Analysis of Antigen-Specific Memory B Cell Antibody Repertoires in PBMCs
Source: Front Immunol. 2019 Jun 25;10:1452. doi: 10.3389/fimmu.2019.01452 (PMC6603168; doi:10.3389/fimmu.2019.01452)
Supplement: Supplementary file 4 [file Data_Sheet_3.PDF]

SUPPLEMENTARY IMAGE S3. Light chain germline V-gene usage among PBMC and Stimulated PBMC repertoires.

Plots show light chain V-gene usage for one replicate of each of the 3 donor repertoires (A: 147, B: 536 and C: 682) sampled at the August 2018 timepoint. Plots are made using the Alakazam R scripts in the Immcantation pipeline.

(A) 147

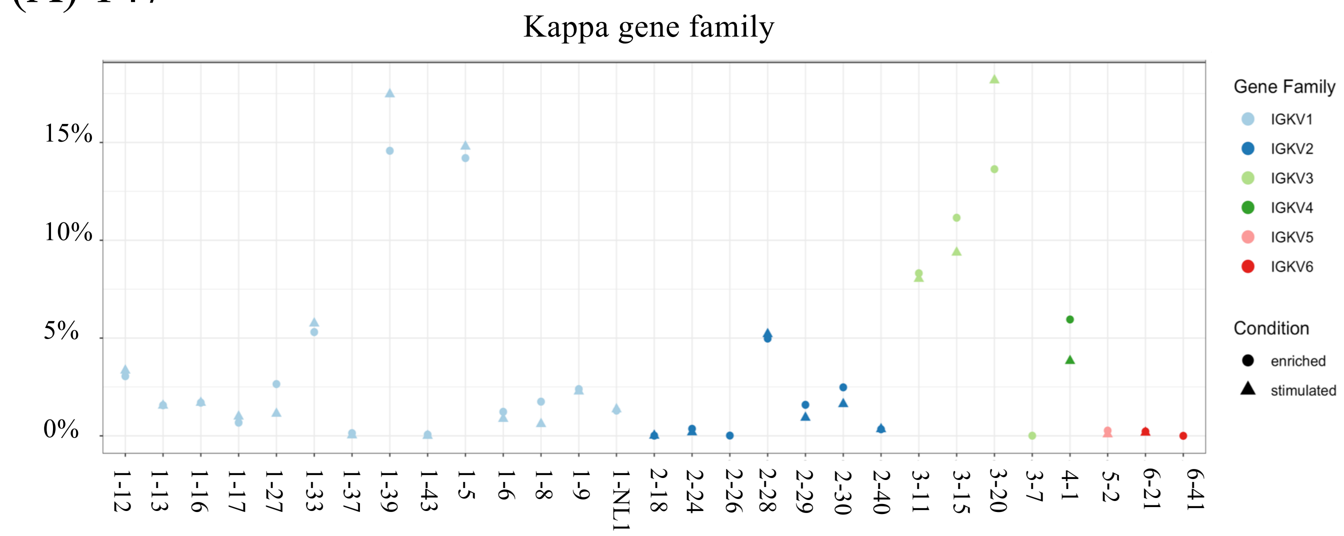

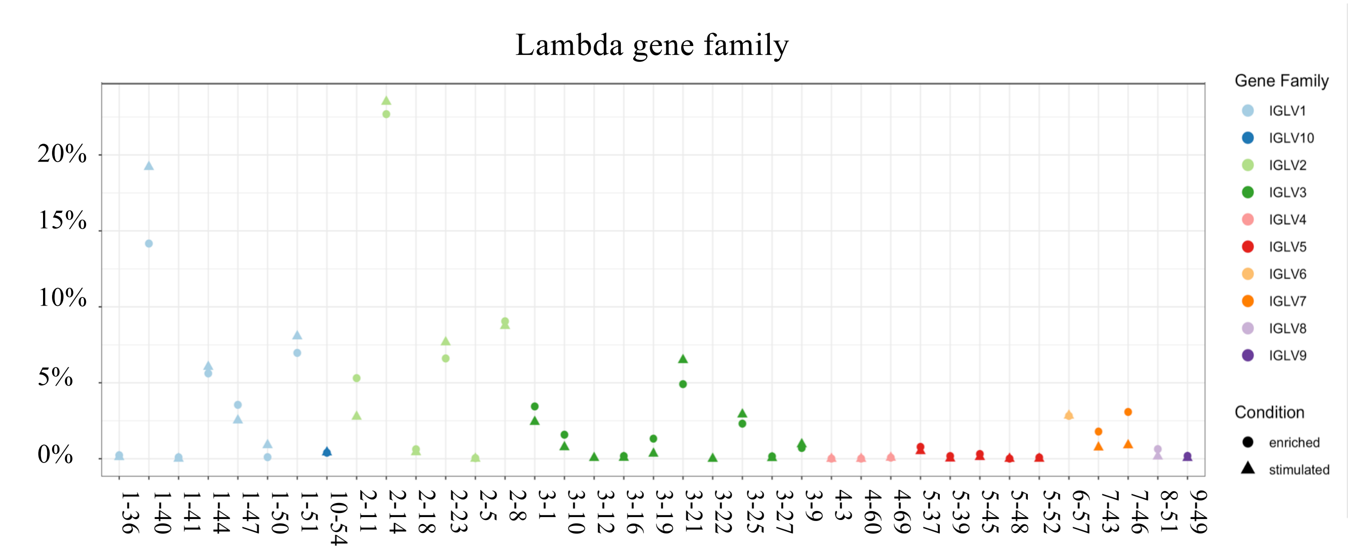

(B) 536

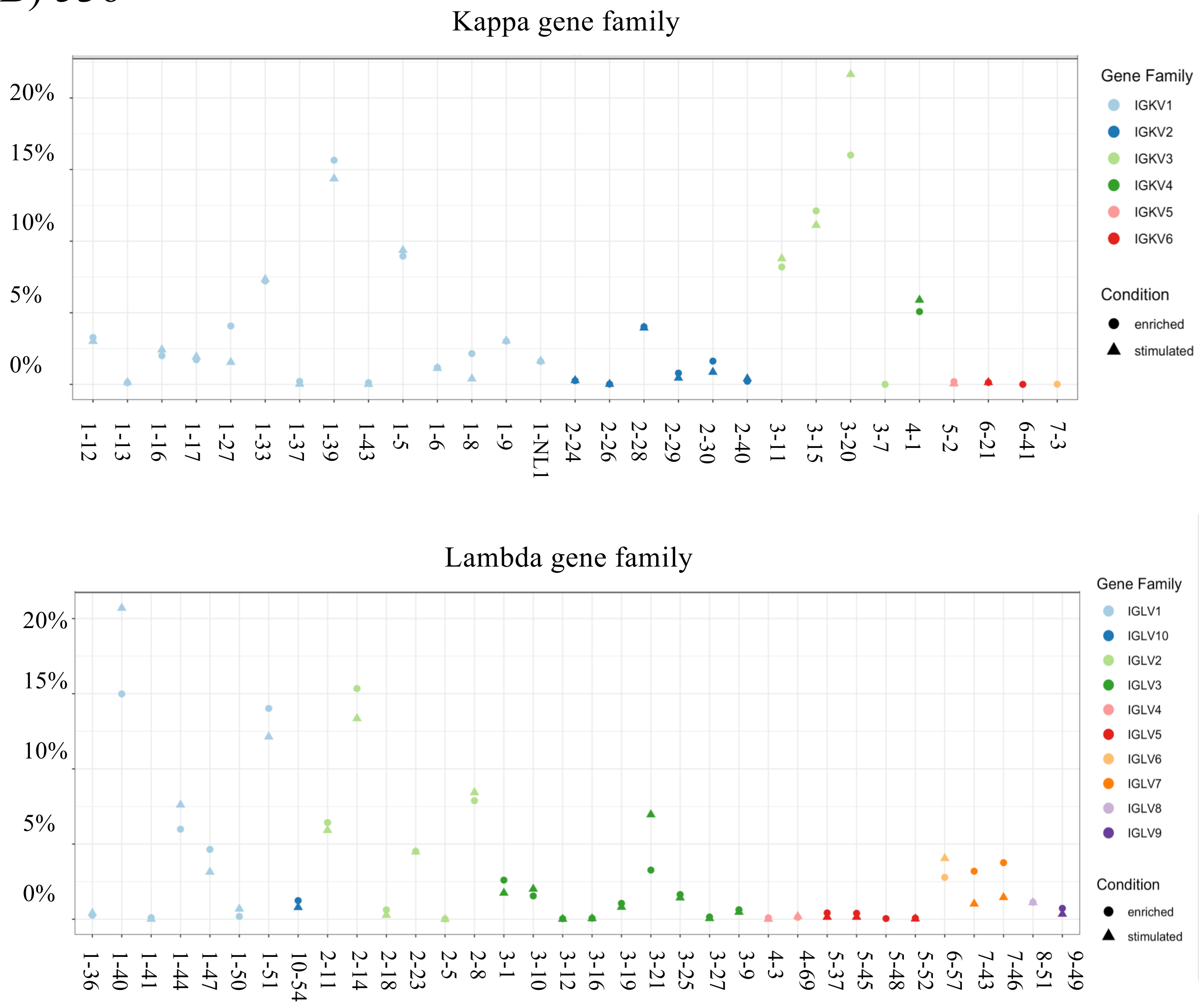
Kappa gene family
20%
15%
10%
5%
0%
1-12 1-13 1-16 1-17 1-27 1-33 1-37 1-39 1-43 1-5 1-6 1-8 1-9 1-NL1 2-24 2-26 2-28 2-29 2-30 2-40 3-11 3-15 3-20 3-7 4-1 5-2 6-21 6-41 7-3
Gene Family
IGKV1
IGKV2
IGKV3
IGKV4
IGKV5
IGKV6
Condition
enriched
stimulated
Lambda gene family
20%
15%
10%
5%
0%
1-36 1-40 1-41 1-44 1-47 1-50 1-51 10-54 2-11 2-14 2-18 2-23 2-5 2-8 3-1 3-10 3-12 3-16 3-19 3-21 3-25 3-27 3-9 4-3 4-69 5-37 5-45 5-48 5-52 6-57 7-43 7-46 8-51 9-49
Gene Family
IGLV1
IGLV10
IGLV2
IGLV3
IGLV4
IGLV5
IGLV6
IGLV7
IGLV8
IGLV9
Condition
enriched
stimulated

(C) 682

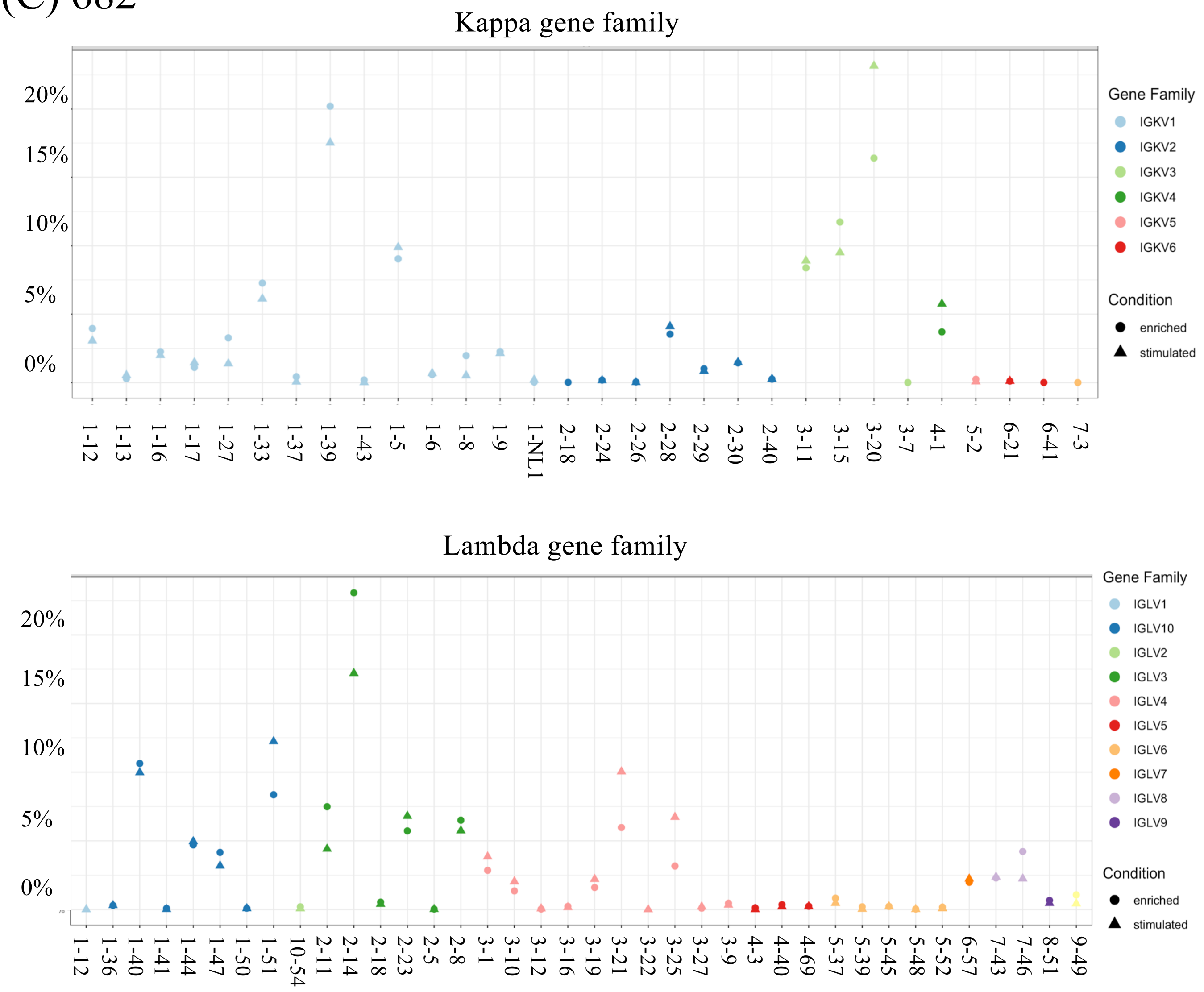
Kappa gene family
20%
15%
10%
5%
0%
1-12
1-13
1-16
1-17
1-27
1-33
1-37
1-39
1-43
1-5
1-6
1-8
1-9
1-NL1
2-18
2-24
2-26
2-28
2-29
2-30
2-40
3-11
3-15
3-20
3-7
4-1
5-2
6-21
6-41
7-3
Gene Family
IGKV1
IGKV2
IGKV3
IGKV4
IGKV5
IGKV6
Condition
enriched
stimulated
Lambda gene family
20%
15%
10%
5%
0%
1-12
1-36
1-40
1-41
1-44
1-47
1-50
1-51
10-54
2-11
2-14
2-18
2-23
2-5
2-8
3-1
3-10
3-12
3-16
3-19
3-21
3-22
3-25
3-27
3-9
4-3
4-40
4-69
5-37
5-39
5-45
5-48
5-52
6-57
7-43
7-46
8-51
9-49
Gene Family
IGLV1
IGLV10
IGLV2
IGLV3
IGLV4
IGLV5
IGLV6
IGLV7
IGLV8
IGLV9
Condition
enriched
stimulated
